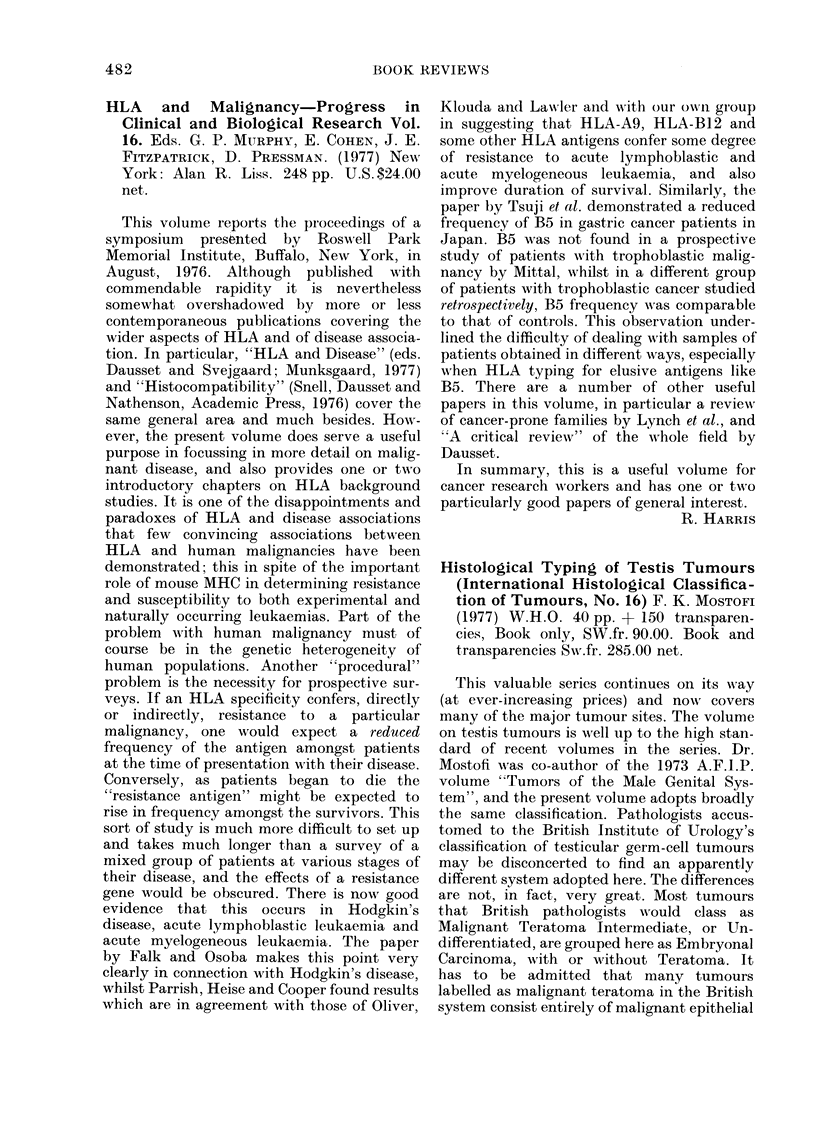# HLA and Malignancy—Progress in Clinical and Biological Research Vol. 16

**Published:** 1978-03

**Authors:** R. Harris


**HLA and Malignancy—Progress in Clinical and Biological Research Vol. 16.** Eds. G. P. MURPHY, E. COHEN, J. E. FITZPATRICK, D. PRESSMAN. (1977) New York: Alan R. Liss. 248 pp. U.S.$24.00 net.

This volume reports the proceedings of a symposium presented by Roswell Park Memorial Institute, Buffalo, New York, in August, 1976. Although published with commendable rapidity it is nevertheless somewhat overshadowed by more or less contemporaneous publications covering the wider aspects of HLA and of disease association. In particular, "HLA and Disease" (eds. Dausset and Svejgaard; Munksgaard, 1977) and "Histocompatibility" (Snell, Dausset and Nathenson, Academic Press, 1976) cover the same general area and much besides. However, the present volume does serve a useful purpose in focussing in more detail on malignant disease, and also provides one or two introductory chapters on HLA background studies. It is one of the disappointments and paradoxes of HLA and disease associations that few convincing associations between HLA and human malignancies have been demonstrated; this in spite of the important role of mouse MHC in determining resistance and susceptibility to both experimental and naturally occurring leukaemias. Part of the problem with human malignancy must of course be in the genetic heterogeneity of human populations. Another "procedural" problem is the necessity for prospective surveys. If an HLA specificity confers, directly or indirectly, resistance to a particular malignancy, one would expect a *reduced* frequency of the antigen amongst patients at the time of presentation with their disease. Conversely, as patients began to die the "resistance antigen" might be expected to rise in frequency amongst the survivors. This sort of study is much more difficult to set up and takes much longer than a survey of a mixed group of patients at various stages of their disease, and the effects of a resistance gene would be obscured. There is now good evidence that this occurs in Hodgkin's disease, acute lymphoblastic leukaemia and acute myelogeneous leukaemia. The paper by Falk and Osoba makes this point very clearly in connection with Hodgkin's disease, whilst Parrish, Heise and Cooper found results which are in agreement with those of Oliver, Klouda and Lawler and with our own group in suggesting that HLA-A9, HLA-B12 and some other HLA antigens confer some degree of resistance to acute lymphoblastic and acute myelogeneous leukaemia, and also improve duration of survival. Similarly, the paper by Tsuji *et al.* demonstrated a reduced frequency of B5 in gastric cancer patients in Japan. B5 was not found in a prospective study of patients with trophoblastic malignancy by Mittal, whilst in a different group of patients with trophoblastic cancer studied *retrospectively*, B5 frequency was comparable to that of controls. This observation underlined the difficulty of dealing with samples of patients obtained in different ways, especially when HLA typing for elusive antigens like B5. There are a number of other useful papers in this volume, in particular a review of cancer-prone families by Lynch *et al.*, and "A critical review" of the whole field by Dausset.

In summary, this is a useful volume for cancer research workers and has one or two particularly good papers of general interest.

R. HARRIS